# 3D Ordered Macroporous Mn, Zr‐Doped CaCO_3_ Nanomaterials for Stable Thermochemical Energy Storage

**DOI:** 10.1002/advs.202412082

**Published:** 2024-12-16

**Authors:** Han Li, Jinfeng Lin, Jianze Wu, Jiashun Wang, Pengzhao Wang, Guojian Kang, Shuping Huang, Mingkai Fu, Jinjia Wei, Zhengxin Ding, Jinlin Long

**Affiliations:** ^1^ State Key Lab of Photocatalysis on Energy and Environment Fuzhou University Fuzhou 350116 P. R. China; ^2^ College of Chemistry Fuzhou University Fuzhou 350116 P. R. China; ^3^ College of Chemical Engineering Fuzhou University Fuzhou 350116 P. R. China; ^4^ Institute of Electric Engineering Chinese Academy of Sciences Beijing 100190 P. R. China; ^5^ College of Chemical Engineering Xi'an Jiaotong University Xi'an 710049 P. R. China

**Keywords:** CaCO_3_, Ca‐looping, ordered macropores, photonic crystals, thermochemical energy storage

## Abstract

Developing high‐performance Ca‐based materials that can work for long‐term heat transfer and storage in concentrated solar power plants is crucial to achieve the large‐scale conversion of solar photon fluxes to dispatchable electricity. This work demonstrates that a series of Mn, Zr co‐doped CaCO_3_ nanomaterials with the 3D ordered macroporous (3DOM) skeletons are successfully prepared by a novel strategy of templated metal salt co‐precipitation. The characterization results indicate that a majority of Zr and Mn are atomically dispersed into the highly‐crystallized CaCO_3_ framework, whereas a minor amount of Mn is present in the form of CaMnO_3_ nanoparticles (NPs). The optimal 3DOM material made by templating with PS microspheres with a diameter of ≈350 nm, 3DOM‐Ca80Mn10Zr10, shows a solar light absorptance of ≈74.1% and an initial energy storage density of 1706.4 kJ kg^−1^. Importantly, it gives an impressive energy storage density loss of < 6.0% and maintains above 1600 kJ kg^−1^ after 125 cycles. The density functional theory calculations reveal that the co‐doping of Mn and Zr into the CaO crystal lattice offers a strong affinity to [Ca_4_O_4_] clusters, as a result, the anti‐sintering of CaO NPs is significantly enhanced under high temperature.

## Graphical abstract

Mn, Zr co‐doped CaCO_3_ nanoarchitectures with 3D ordered macroporous (3DOM) skeletons are prepared by templated metal salt co‐precipitation for heat transfer and storage in concentrated solar power. The 3DOM structure enhances the solar absorbance and CO_2_ diffusion. Co‐doping of Mn, and Zr into the crystal lattice of CaCO_3_ skeletons offers a strong affinity to CaO nanoparticles, suppressing its sintering.

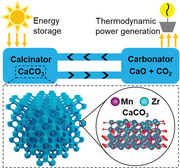

## 1 Introduction

Beyond solar photovoltaics (SPV) is a direct pathway to covert the diffuse solar photon fluxes to electricity, concentrated solar power (CSP) plant with thermochemical energy storage (TCES) system is emerging as a second‐generation technology that converts solar irradiation to high‐temperature heat at a low‐cost and large‐scale, and then dispatchable electricity via a steam turbine (Rankine cycle).^[^ ^1^ ^]^ The TCES system corrects the mismatch between the unsteady solar supply and the electricity demand via a heat transfer and storage medium such as solid media (e.g., regenerator storage), pressurized water (or Ruths storage), molten salt, latent heat, and thermochemical media.^[^ ^2^ ^]^ Although the molten salt‐based TCES systems operated at a low temperature of 550 °C were commercially available, much effort was devoted to develop calcium‐looping (CaL) TCES system that is based on the reversible calcination/carbonation reaction of CaCO_3_(s) ↔ CaO(s) + CO_2_(g) (Equation S1, Supporting Information, ΔH = 178 kJ mol^−1^) for large‐scale CSP applications.^[^ ^3^ ^]^ The CaCO_3_ decomposition commonly proceeds at a high reaction temperature of 600–900 °C.^[^ ^4^ ^]^ By its reverse reaction, the corresponding enthalpy change (ΔH) of −178 kJ mol^−1^ is released to enable a higher energy storage efficiency for power generation. Nevertheless, natural, pure CaCO_3_ does not match with the unique requirements of the CaL‐CSP plants on light absorption and thermal stability, in despite of its non‐toxic, non‐corrosive, and abundant earth. The development of high‐performance Ca‐based materials that can work for long‐term heat transfer and storage was a challenging task for large‐scale CaL‐CSP applications.

In view of the carbonation reaction kinetics, the carbonation step of CaO to CaCO_3_ is most crucial for the CaL operation. Gaseous CO_2_ rapidly diffuses to the surface of CaO particles and slowly penetrates from the surface to the interior for the reaction.^[^ ^5^ ^]^ Unfortunately, because CaO has a low Tammann temperature of ≈530 °C,^[^ ^6^ ^]^ the atomic‐scale calcium migration and agglomeration are easily proceeded upon calcination and carbonation at a high operating temperature of 600–900 °C.^[^ ^7^ ^]^ It makes pores clogged, forming highly‐dense CaCO_3_ thin‐layer on the surface of CaO particles, as a result, the CO_2_ diffusion to the interior is greatly retarded, even ceased. Correspondingly, the energy storage density is rapidly declined after multiple cycles of repeated calcination and carbonation as the surface area and pore volume are significantly decreased.^[^ ^7^ ^]^ Grasa and Abanades studied the CO_2_ capture capacity of various natural limestone in a long series of carbonation/calcination cycles,^[^ ^8^ ^]^ and demonstrated an initial conversion ratio of ≈75% for the carbonation reaction. However, a rapid decay to 27.3% was occurred after the 11 cycles. Two strategies have been developed to address the scientific issue of cycling instability of CaO/CaCO_3_ for heat transfer and storage. One was the preparation of nanoscale porous Ca‐based materials with a large initial surface area and rich porous structure, which promoted the carbonation/calcination cycle reactions. Several classical preparation methods including sol‐gel, co‐precipitation, flame‐spray pyrolysis, hydrothermal, template, atomic layer deposition, recrystallization, and bubbling were employed to do that.^[^ ^9^ ^]^ The activity and stability of as‐prepared resultants were greatly better than those of natural CaCO_3_ particles. Taking cage‐like CaO hollow microspheres prepared by a template‐assisted approach as an example,^[^ ^10^ ^]^ even if under a harsh condition of 650 °C, an initial conversion of 82.5% was achieved within 15 min at an atmosphere of 15 vol.% CO_2_ and 85 vol.% N_2_ for the carbonation reaction, higher than that (63.2%) of the reference limestone. After 15 cycles, they still possessed a carbonation value of 39.7%, exceeding the reference limestone (16.0%) by 148.1%. Another was the introduction of one or more inert components (Mn, Mg, Co, Al, Zr, Ti, Cr, Ni, La, Y, Zn, Si, Ce, Nd, Li, and Ba) with a higher Tammann temperature. The metal incorporation was able to not only inhibit the calcium agglomeration and sintering at high temperature to alleviate clogging of material pores,^[^ ^9^, ^11^ ^]^ but also significantly enhance the optical absorption of CaO/CaCO_3_ by one or more dark components, especially Mn.^[^ ^9^, ^12^ ^]^ Many studies showed that introducing Mn into CaO/CaCO_3_ led to the formation of black manganate compounds such as Ca_4_Mn_3_O_10_ and CaMnO_3_.^[^ ^13^ ^]^ Besides the improvement of solar absorption capacity, they also acted as a “spacer” for stabilizing CaO nanoparticles (NPs) by the strong interaction occurring at the interface of CaO/Ca_4_Mn_3_O_10_ and CaO/CaMnO_3_, and yet the energy storage density was greatly decreased.^[^ ^14^ ^]^ The kinetic considerations meant that designing and constructing easily accessible interconnected pore channels for the Ca‐based materials were necessary for excluding the diffusion‐controled limitation and eventually enhancing the rates of carbonation/calcination cycle reactions and the TCES stability, and yet little work has been done for this target up to date.

Herein, a novel strategy of templated metal salt co‐precipitation was developed to prepare a series of 3D ordered macroporous (3DOM) Mn, Zr co‐doped CaCO_3_ materials via templating with polystyrene (PS) microspheres. The templated co‐precipitation of low‐valent (Ca^2+^ and Mn^2+^) and high‐valent (Zr^4+^) metal acetates in the interstices of the colloid crystals and subsequent chemical conversion of metal acetates to oxalates by the ligand exchange method. The resulting 3DOM Mn, Zr co‐doped CaCO_3_ nanomaterials possessed a periodic macroporous structure and a mesoporous skeleton. The skeletal thickness was controlled in the region of 30–100 nm via changing concentrations of Mn and Zr dopants in the mixed precursor solution. The characterization results indicated that a majority of Mn and Zr were atomically dispersed into the highly‐crystallized CaCO_3_ framework, whereas a minor amount of Mn was present in the form of CaMnO_3_ NPs. The optimal 3DOM‐CaCO_3_ material made by templating with PS microspheres with a diameter of ≈350 nm gave an average pore diameter of ≈250 nm for improving the solar light absorption. As a result, a 4 fold enhanced light absorption at the spectral region of 200–2500 nm was obtained compared to the commercial pure CaCO_3_ material, and the as‐synthesized Mn, Zr co‐doped CaCO_3_ material denoted as 3DOM‐Ca80Mn10Zr10 showed a solar light absorption of ≈74.1% and an initial energy storage density of 1706.4 kJ kg^−1^. Amazingly, the 3DOM Ca80Mn10Zr10 material gave an impressive energy storage density loss of < 6.0% and maintained above 1600 kJ kg^−1^ after 125 cycles, whereas the reference already lost the energy storage density of > 14.0% after 60 cycles. The results indicated conclusively that the highly ordered macroporous/mesoporous structure endowed a larger rate of CO_2_ diffusion and penetration into the Ca‐based skeletons, significantly improving the energy storage density. The density functional theory (DFT) calculations indicated that the co‐doping of Mn and Zr into the [100] plane of CaO NPs led to a large adsorption energy of the [Ca_4_O_4_] clusters, which was increased with the dopant concentrations. The strong interaction offered a predominant contribution to suppress the sintering of CaO NPs under high temperature. This work opened a new avenue to break the bottleneck of Ca‐based materials for energy storage applications.

## 2 Results and Discussion

### 2.1 Synthesis and Characterizations

**Figure 1:**
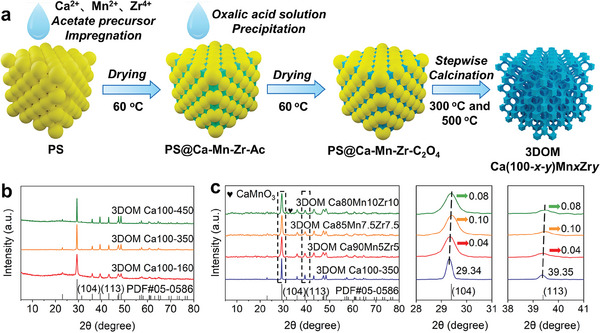
a) Synthetic procedure of 3DOM Ca‐based materials, b) XRD patterns of as‐synthesized pure 3DOM‐Ca100 materials, and c) XRD patterns of as‐synthesized 3DOM Mn, Zr co‐doped Ca‐based materials.

The synthetic procedure of 3DOM Ca‐based materials is depicted in **Figure** **1**a. To begin with, the monodisperse PS microspheres with an average diameter of ≈160, 350, and 450 nm were self‐assembled to the 3D ordered photonic crystal templates in water (Figures S1 and S2, Supporting Information).^[^ ^17^ ^]^ Following this, the inorganic precursors with different molar ratios of Ca, Mn, and Zr acetate salts dissolved into a mixed alcohol/water solution were introduced into the voids of the PS photonic crystal templates and then were dried at 60 °C for 8 h. The resulting solids denoted as PS@Ca‐Mn‐Zr‐Ac were immerged into an oxalic acid solution for in situ chemical conversion to metal oxalates, and then was dried at 60 °C for 8 h. Finally, the resultant PS@Ca‐Mn‐Zr‐C_2_O_4_ was stepwise calcinated at 300 °C for 2 h and 500 °C for 2 h to completely remove the PS templates. The as‐synthesized Ca‐based materials were denoted as 3DOM Ca(100‐x‐y)MnxZry (x and y represented, respectively, the molar contents of Mn and Zr dopants). The molar contents of Mn and Zr doped in the fresh 3DOM CaCO_3_ materials were determined by inductively coupled plasma optical emission spectroscopy (ICP‐OES) and listed in Table S1 (Supporting Information). The results showed that the molar ratios of Ca, Mn, and Zr elements in the resultant solids were almost uniform with the feed ratios.

Following the above‐mentioned methodology, three pure 3DOM Ca100 samples with the 160, 350, and 450 nm PS templates were first synthesized to explore the optimal conditions. The scanning electron microscopy (SEM) images of as‐synthesized pure CaCO_3_ materials (Figure S3, Supporting Information) confirmed the formation of the well‐defined 3DOM structure after removing the PS templates for the two 3DOM Ca100‐350 and −450 samples, whereas the 3DOM structure was ill‐defined using the 160 nm PS template. Figure 1b displays the X‐ray powder diffraction (XRD) patterns of as‐synthesized 3DOM Ca100‐160, −350, and −450 samples. All Bragg diffraction peaks were well indexed to the hexagonal calcite CaCO_3_ (space group R‐3c(167), JCPDS card #05‐0586), without any other phases. Moreover, the BET area of the 3DOM Ca100‐350 sample was larger than those of the 3DOM Ca100‐450 sample (Table S2, Supporting Information), indicating that the 350 nm PS template was optimal to prepare Mn, Zr co‐doped 3DOM CaCO_3_ materials. By changing the Ca/Mn/Zr feed ratios, a series of 3DOM Ca(100‐x‐y)MnxZry materials can be obtained. Figure 1c showed the XRD patterns of three representative Mn, Zr co‐doped 3DOM CaCO_3_ samples, 3DOM Ca90Mn5Zr5, 3DOM Ca85Mn7.5Zr7.5, and 3DOM Ca80Mn10Zr10. The co‐doping of Mn and Zr did not alter the crystal phase of CaCO_3_, but made the Bragg diffraction peaks at 2θ = 29.34° and 39.35° indexed, respectively, to the (104) and (113) planes of calcite CaCO_3_ shifted toward high angle. The shift of the (104) and (113) diffraction peaks was increased with an increase in dopant content and reached a maximum at a molar doping content of 7.5% for Mn and Zr, indicating clearly that both of Mn and Zr were co‐incorporated into the crystal lattice of CaCO_3_. According to the Bragg equation of *d* = λ/2sinθ, the crystal cell parameters were calculated and listed in Table S3 (Supporting Information). The interplanar spacings of the (104) and (113) planes were equal, respectively, to be 3.0416 and 2.2870 Å for 3DOM Ca100‐350, 3.0376 and 2.2848 Å for 3DOM Ca90Mn5Zr5, 3.0315 and 2.2814 Å for 3DOM Ca85Mn7.5Zr7.5, and 3.0324 and 2.2826 Å for 3DOM Ca80Mn10Zr10. As a result, the crystal cell volume (V) was increased gradually from 369.43 Å^3^ for 3DOM Ca100‐350, to 368.32 Å^3^ for 3DOM Ca90Mn5Zr5, to 366.28 Å^3^ for 3DOM Ca85Mn7.5Zr7.5, and to 366.70 Å^3^ for 3DOM Ca80Mn10Zr10, because the ionic radiuses of Mn^2+^ (0.067 nm) and Zr^4+^ (0.072 nm) dopants were smaller than that (0.099 nm) of Ca^2+^.^[^ ^15^ ^]^ The results further confirmed that Ca^2+^ ions in the CaCO_3_ skeletons were replaced by Mn^2+^ and Zr^4+^ dopants.

Notably, a weak diffraction peak belonging to the (220) plane of cubic perovskite CaMnO_3_ (space group Pm‐3m(221), JCPDS card 03–0830) occurred at 2θ = 33.92° when the molar contents of Mn and Zr dopants were increased to 10.0%. It was ascribed to the decomposition of Ca(1‐x)MnxCO_3_ formed by the lattice doping of excessive Mn atoms in CaCO_3_ under high‐temperature calcination.^[^ ^16^ ^]^ Such CaMnO_3_ particles were highly stable and dispersed into the 3DOM CaCO_3_ skeletons, and thus served as a “spacer” to suppress the sintering of CaO NPs in long series of carbonation/calcination cycles. No zirconium oxides and zirconate compounds were observed from the XRD pattern of 3DOM Ca80Mn10Zr10 after the stepwise calcination at 300 °C for 2 h and 500 °C for 2 h, suggesting that Zr was not separated from the crystal lattice of CaCO_3_ and it was uniformly dispersed into the 3DOM CaCO_3_ skeletons.

**Figure 2:**
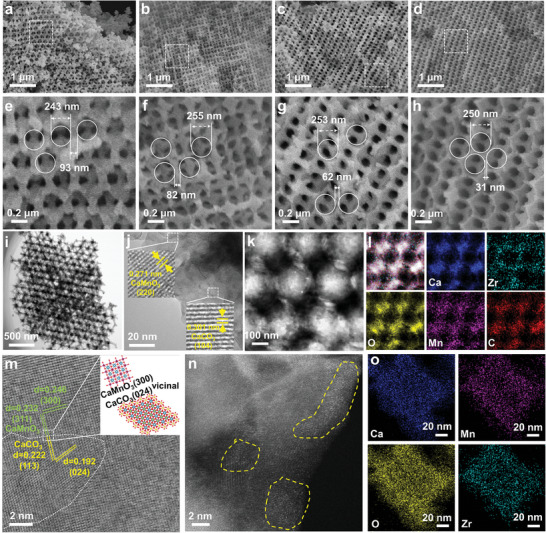
SEM images of as synthesized 3DOM Ca‐based samples. a,e) 3DOM Ca100‐350, b,f) 3DOM Ca90Mn5Zr5, c,g) 3DOM Ca85Mn7.5Zr7.5, and d,h) 3DOM Ca80Mn10Zr10. i,j) TEM and HRTEM images of fresh 3DOM Ca80Mn10Zr10. k,l) HAADF‐STEM and EDX elemental mapping images of fresh 3DOM Ca80Mn10Zr10. m,n) aberration‐corrected HRTEM and HAADF‐STEM images of fresh 3DOM Ca80Mn10Zr10. o) high‐resolution EDX elemental mapping images of fresh 3DOM Ca80Mn10Zr10.

The shape and morphology of Mn, Zr co‐doped 3DOM CaCO_3_ samples were visualized with scanning electron microscopy (SEM). As shown in **Figure** **2**, the highly‐ordered macropores were well‐arranged to form interconnected channels for the four 3DOM samples, and the highly cross‐interconnected macropores possessed an unfirm diameter of 250 ± 5 nm. Interestingly, some visible changes in the 3DOM CaCO_3_ skeletons were observed by altering the molar ratios of Ca, Mn, and Zr. The skeletal thickness was decreased from ≈93 nm for the 3DOM Ca100‐350 sample, to ≈82 nm for the 3DOM Ca90Mn5Zr5 sample, to ≈62 nm for the 3DOM Ca85Mn7.5Zr7.5 sample, and to ≈31 nm for the 3DOM Ca80Mn10Zr10 sample. Larger the doping contents of Mn and Zr, thinner the 3DOM skeletal thickness. It indicated that the doping of inert components inhibited the sintering of CaCO_3_ NPs in the 3DOM skeleton under high‐temperature calcination. As a result, the 3DOM CaCO_3_ skeleton became to be thinner and thinner with an increase in Mn and Zr content.

Next, transmission electron microscopy (TEM) and high‐resolution TEM (HRTEM) were applied to clarify the crystal phases and compositions of the 3DOM CaCO_3_ skeletons. As displayed in Figure 2i,j representing the TEM and HRTEM images of the 3DOM Ca80Mn10Zr10 sample, it was clearly observed that the lattice fringe of 0.301 nm was corresponded to the (104) plane of CaCO_3_ and the lattice fringe of 0.271 nm was corresponded to the (220) plane of CaMnO_3_. The HAADF‐STEM and EDX elemental mapping images (Figure 2k,l) confirmed that Mn and Zr elements were homogeneously distributed into the 3DOM CaCO_3_ skeletons. It appeared from the aberration‐corrected HRTEM image shown in Figure 2m that CaMnO_3_ NPs were buried into the CaCO_3_ skeletons and there existed a clear interface between the (300) plane of CaMnO_3_ and the (024) plane of CaCO_3_. The aberration‐corrected HAADF‐STEM image (Figure 2n) showed many bright dots belonging to Zr atoms and thus offered solid evidences for the conclusion that Zr was atomically incorporated into the crystal lattice of CaCO_3_. The EDX elemental mapping images (Figure 2o) further confirmed that Zr and Mn were highly dispersed into the 3DOM CaCO_3_ skeletons.

**Figure 3:**
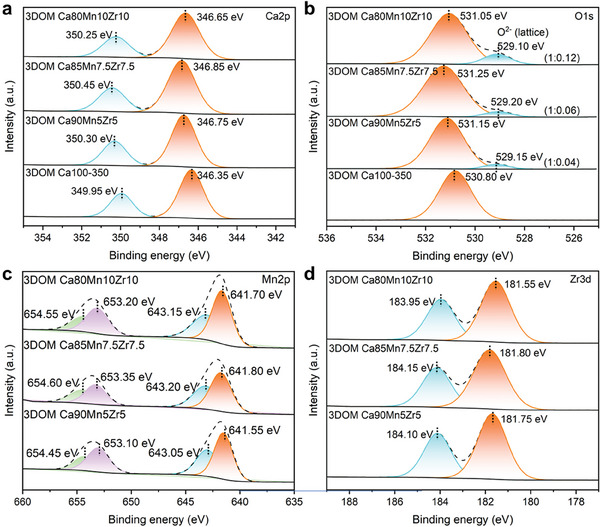
XPS spectra of as‐synthesized 3DOM Ca‐based samples, a) Ca2p; b) O1s; c) Mn2p; d) Zr3d.

The chemical states of dopants were determined by X‐ray photoelectron spectroscopy (XPS). As displayed in **Figure** **3**a, the binding energies of Ca2p_1/2_ and Ca2p_3/2_ were 349.95 and 346.35 eV, respectively, derived from Ca^2+^ ions in CaCO_3_. After the co‐doping of Mn and Zr, the Ca2p binding energies were shifted toward high energy, indicating that the strong electronic interaction between Ca^2+^ ions and dopants. Expectedly, besides the O1s peak at ≈531.0 eV belonging to the O atoms of CO_3_^2−^ anions (Figure 3b), a new O1s peak occurred at ≈529.15 eV in the O1s XPS spectra after the co‐doping of Mn and Zr, and the peak intensity was much low, and yet increased with the content of Mn dopants. Together with the above‐mentioned XRD and TEM results, it was concluded that the O1s peak at ≈529.15 eV was contributed from the lattice oxygen of CaMnO_3_. Also, the dopant‐induced binding energy shift was observed in the Mn2p XPS spectra of three Mn, Zr co‐doped CaCO_3_ samples (Figure 3c), highly identical with Ca2p. The Mn2p_3/2_ XPS spectra were deconvoluted into two fitted peaks located at 643.05 and 641.55 eV. The former was corresponded to Mn^4+^ and the latter was attributed to Mn^3+^,^[^ ^17^ ^]^ demonstrating that Mn^2+^ was oxidized to Mn^3+^ and Mn^4+^ during the high‐temperature calcination. The Zr3d XPS spectra (Figure 3d) showed a Zr3d_3/2_ peak at 184.10 eV and a Zr3d_5/2_ peak at 181.75 eV, attributed to Zr^4+^.^[^ ^18^ ^]^ Interestingly, the Zr 3d binding energy of the 3DOM Ca80Mn10Zr10 sample was shifted by 0.2 eV toward low energy in comparison with those of the 3DOM Ca90Mn5Zr5 sample, suggesting that the strong electronic interaction led to a significant increase in electron density of Zr atoms doped into the crystal lattice of CaCO_3_, and a reverse decrease in electron density of Ca and co‐doped Mn atoms. These results indicated that the co‐doping of Mn and Zr into the crystal lattice of CaCO_3_ generated a powerful force to draw electrons from adjacent CaCO_3_, greatly beneficial to suppress the sintering of CaCO_3_ NPs.

**Figure 4:**
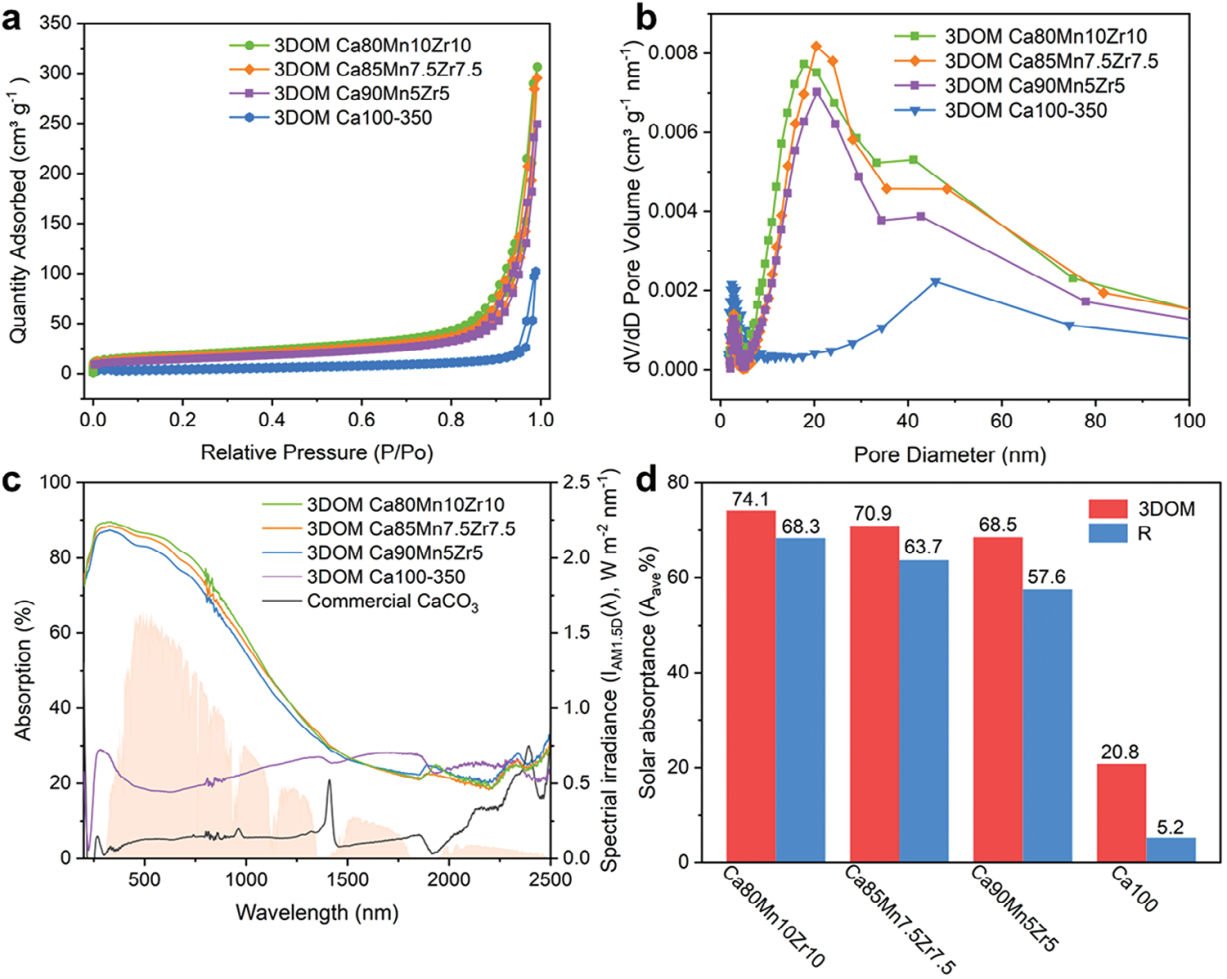
a) N_2_ adsorption‐desorption isotherms at 77 K of 3DOM Ca100‐350, Ca90Mn5Zr5, Ca85Mn7.5Zr7.5, and Ca80Mn10Zr10. b) Pore size distribution of 3DOM Ca100‐350, Ca90Mn5Zr5, Ca85Mn7.5Zr7.5, and Ca80Mn10Zr10. c) UV–vis–NIR diffuse‐reflection spectra of commercial CaCO_3_, 3DOM Ca100‐350, Ca90Mn5Zr5, Ca85Mn7.5Zr7.5, and Ca80Mn10Zr10. d) Comparison of average solar absorptance with the reference samples.

Then, the specific surface area and pore volume of all as‐synthesized 3DOM CaCO_3_ samples were determined by N_2_ adsorption‐desorption at 77 K. As shown in **Figure** **4**a and listed in Table S2 (Supporting Information), all 3DOM samples exhibited a typical type III isotherm, and the Brunauer‐Emmett‐Teller (BET) surface area increased with the content of Mn and Zr dopants. The 3DOM Ca80Mn10Zr10 sample gave a 4 fold improved BET surface area of 65.5 m^2^ g^−1^ compared to the 3DOM Ca100‐350 sample. The pore size distribution of 3DOM Ca100‐350 sample was centered at ≈50 nm (Figure 4b), whereas the three 3DOM Ca80Mn10Zr10, Ca85Mn7.5Zr7.5, and Ca90Mn5Zr5 samples exhibited a regular aperture distribution centered at ≈20 nm with a shoulder centered at ≈50 nm. It indicated that the co‐doping of Mn and Zr into the crystal lattice of CaCO_3_ skeletons generated a large amount of mesopores in the 3DOM structure, highly beneficial to the carbonation reaction.

The light absorptance in the wavelength range of 200–2500 nm of as‐synthesized 3DOM CaCO_3_ samples was studied by the UV–vis–NIR spectrophotometer, as shown in Figure 4c,d. The spectral absorptance of pure commercial CaCO_3_ was low, with a calculated average solar absorptance of 5.2%, while a 4 fold enhanced absorptance, 20.8%, was obtained for the 3DOM Ca100‐350 sample. The Mn, Zr co‐doping enhanced significantly the light absorptance of CaCO_3_ skeletons, which increased with the content of dopants. The 3DOM Ca80Mn10Zr10 sample gave a light absorptance of 74.1%, higher than that (68.3%) of the control sample synthesized by the sol‐gel method, Ca80Mn10Zr10‐R (Figure 4d). Another two doped samples, 3DOM Ca85Mn7.5Zr7.5 and 3DOM Ca90Mn5Zr5, showed, respectively, a light absorptance of 70.1% and 68.5%, superior to their corresponding control samples, Ca85Mn7.5Zr7.5‐R (63.7%) and Ca90Mn5Zr5‐R (57.6%) (Figure S5d, Supporting Information). These results showed that the periodic arrangement of macropores into 3DOM structure (i.e., photonic crystal structure^[^ ^19^ ^]^) efficiently improve the light absorption properties of the Ca‐based materials. The presence of Bragg reflection peaks centered at ≈480 nm (Figure S5, Supporting Information), which was highly consistent with the results reported in literature.^[^ ^19c–e^ ^]^ The little increase of 5.8% in light absorptance may be originated from modulation of light by the 3DOM structure. Thus, the 3DOM structure fulfilled the requirement for effective utilization of sunlight in the calcium‐based energy storage materials.

### 2.2 Thermochemical Energy Storage Performance

**Figure 5:**
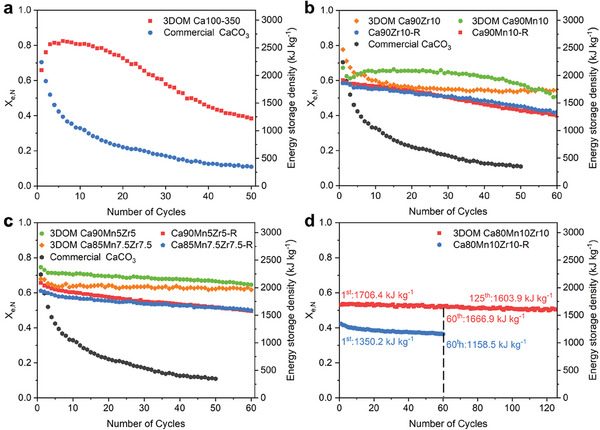
a) Cyclic TCES performance of 3DOM Ca100‐350 and commercial CaCO_3_. b) Cyclic TCES performance of 3DOM Ca90Mn10, 3DOM Ca90Zr10, 3DOM Ca90Mn5Zr5, Ca90Mn10‐R, and Ca90Zr10‐R. c) Cyclic TCES performance of 3DOM Ca90Mn5Zr5, 3DOM Ca85Mn7.5Zr7.5, 3DOM Ca80Mn10Zr10, Ca90Mn5Zr5‐R, and Ca85Mn7.5Zr7.5‐R. d) Comparison of TCES performance with 3DOM Ca80Mn10Zr10 and Ca80Mn10Zr10‐R.

The TCES performance of all as‐synthesized 3DOM Ca‐based materials were estimated with a thermogravimetric analyzer (TGA) under a harsh condition of 800 °C and compared to those of control samples, as shown in **Figures** **5** and S6–S11 (Supporting Information). As expected, the 3DOM Ca100‐350 sample exhibited a substantial improvement in TCES performance, compared to the commercial CaCO_3_ NPs (Figure 5a). The 3DOM Ca100‐350 sample had an initial energy storage density of 2096.5 kJ kg^−1^ and an initial conversion ratio (*X*_e,N_) of 65.9%, slightly lower than the commercial CaCO_3_ NPs. The latter gave an initial energy storage density of 2240.2 kJ kg^−1^ and an initial conversion ratio of 70.4%. Notably, the commercial CaCO_3_ NPs began to decay after the first cycle and lost 84.5% of energy storage density after 50 cycles, while the 3DOM Ca100‐350 sample holds 58.3% of energy storage density after 50 cycles. The results confirmed that the interconnected macropores in 3DOM CaCO_3_ allowed CO_2_ to enter more easily the interior from the external surface, facilitating accessibility to the contact points for the carbonation reaction. The hierarchically mesoporous/macroporous structure was effective for alleviating the pore‐clogging and sintering during the Ca‐looping process. In addition, the 3DOM Ca100‐350 sample showed a maximum conversion ratio of 81.5% at the fourth cycle, the energy storage density was reversely increased to 2590.7 kJ kg^−1^. The increase in conversion was possibly resulted from the locally‐microstructural reconstruction of 3DOM CaCO_3_ skeletons during the repeated calcination/carbonation reactions.^[^ ^20^ ^]^

The incorporation of single inert component (Mn or Zr) into the 3DOM CaCO_3_ skeletons to further improve the TCES stability, as shown in Figure 5b. The 3DOM Ca90Mn10 sample holds an initial energy storage density of 2136.9 kJ kg^−1^, while the initial energy storage density of the 3DOM Ca90Zr10 sample was improved to 2470.2 kJ kg^−1^. The former began to decay after 40 cycles, whereas the latter decayed continuously within the first 15 cycles and then was gradually stabilized at 1729.2 kJ kg^−1^. The two control samples, Ca90Mn10‐R and Ca90Zr10‐R, showed a lower initial energy storage density of 1850–1950 kJ kg^−1^ and a linear decrease in conversion ratio. After 60 cycles, ≈27.4% of energy storage density was lost for Ca90Zr10‐R, while the Ca90Mn10‐R sample lost 33.9% of energy storage density. Interestingly, upon co‐doping of Mn and Zr, the stability was further enhanced, despite that the energy storage density was slightly decreased. Taking the 3DOM Ca90Mn5Zr5 sample as an example. It had an energy storage density of 2371.3 kJ kg^−1^, comparable with the 3DOM Ca90Zr10 sample. After 60 cycles, only 13.4% of energy storage density was lost, superior to the control sample, Ca90Mn5Zr5‐R. The latter gave a lower energy storage density of 2087.4 kJ kg^−1^ and 24.6% of energy storage density loss. Compared with the 3DOM Ca90Zr10 and 3DOM Ca90Mn10 samples, the co‐doping sample had a better stability. It was attributed to the synergism of Mn and Zr co‐doped into the 3DOM CaCO_3_ skeletons.

Additionally, the TCES performance of two co‐doping samples with a higher dopant content, 3DOM Ca85Mn7.5Zr7.5 and 3DOM Ca80Mn10Zr10, were also estimated under the same conditions, as shown in Figure 5c,d. The initial conversion ratio (*X*_e,N_) was decreased in the order of 3DOM Ca90Mn5Zr5 (74.6%) > 3DOM Ca85Mn7.5Zr7.5 (67.9%) > 3DOM Ca80Mn10Zr10 (53.7%), but the stability was significantly enhanced with the dopant content. After 60 cycles, the 3DOM Ca85Mn7.5Zr7.5 sample lost 11.9% of energy storage density, while only 2.3% of energy storage density was lost for the 3DOM Ca80Mn10Zr10 sample. All of Mn, Zr co‐doped 3DOM CaCO_3_ samples were superior to their corresponding control samples. Amazingly, after 125 cycles, the energy storage density of 3DOM Ca80Mn10Zr10 decreased by only 95.4 kJ kg^−1^ and remained above 1600 kJ kg^−1^, and only 6.0% of energy storage density was lost. The reference, Ca80Mn10Zr10‐R, showed a lower initial conversion ratio of 42.5% and a larger energy storage density loss of 14.2% after 60 cycles. Moreover, the thermochemical stability of 3DOM Ca80Mn10Zr10 was superior to those of Ca‐based materials reported in literature, as listed in Table S5 (Supporting Information). These results confirmed that the formation of 3DOM structure was one of main contributors to the excellent TCES performance and cyclic stability.

### 2.3 Reaction Kinetics Analysis

**Figure 6:**
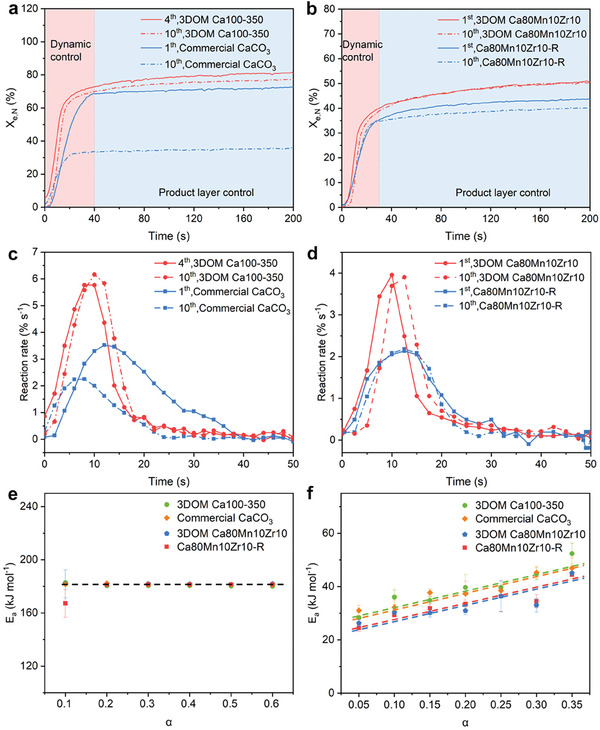
a) the carbonation conversion curves of 3DOM Ca100‐350 and commercial CaCO_3_, b) the carbonation conversion curves of Ca80Mn10Zr10‐R and 3DOM Ca80Mn10Zr10, c) the reaction rate versus time curves of 3DOM Ca100‐350 and commercial CaCO_3_, d) the reaction rate versus time curves of Ca80Mn10Zr10‐R and 3DOM Ca80Mn10Zr10, e) the function of the activation energy of calcination reaction with α values, f) the function of the activation energy of carbonation reaction with α values.

To understand the nature of calcination/carbonation reactions under the isothermal conditions, the reaction kinetics was investigated in detail by performing at the temperature range of 700–800 °C. First, the carbonation reaction kinetics was analyzed under the isothermal thermochemical cycling conditions (800 °C). The carbonation reaction involves two stages: the rapid chemically controlled and diffusion‐limited regimes, i.e., the dynamic control and product layer control stages. For the 3DOM Ca100‐350 sample, the activation stage occurred at the first three cycles as shown in Figure 5a, and thus the fourth cycle of carbonation reaction was used for the kinetic analysis. As shown in **Figure** **6**a, the 3DOM Ca100‐350 sample experienced a shift from the chemically controled to the diffusion‐controled stage within 19 s, quicker than the commercial CaCO_3_ sample (40 s). A maximal conversion rate of 5.92% s^−1^ was achieved at ≈10 s, and yet the peak was occurred at ≈12 s at the 10th cycle (Figure 6c). Moreover, it went on doing the slow carbonation reaction at the product layer control stage, whereas the commercial CaCO_3_ sample almost stopped the carbonation reaction after 40 s. After 10 cycles, the conversion curve of 3DOM Ca100‐350 was hardly changed, but the carbonation conversion ratio (*X*_e,N_) was slightly decreased from 81.5% to 77.4% within 200 s. Differently from 3DOM Ca100‐350, the dynamic control stage of commercial CaCO_3_ was shortened from 40 to 22 s. A maximal carbonation conversion rate of 3.52% s^−1^ occurred at 7 s, and the peak was advanced by 5 s and decreased to 2.36% s^−1^ at the 10th cycle (Figure 6c). In addition, a tremendous decrease in conversion rate was occurred after 10 cycles because of the rapid sintering. These kinetic analysis results indicated clearly that the 3DOM structure with a higher specific surface area and larger pore volume was efficient for promoting the carbonation reaction in the two stages of dynamic and product layer control, improving the cyclic stability.

After co‐doping of Mn and Zr into 3DOM CaCO_3_, the kinetic curve of the carbonation reaction was not altered in the dynamic control regime. As shown in Figure 6b,d, A maximal conversion rate of 3.9% s^−1^ at the first cycle occurred at ≈10 s, and shifted to ≈12 s at the 10th cycle, indicating that the 3DOM Ca80Mn10Zr10 sample had the same dynamic decay trajectory as the 3DOM Ca100‐350 sample in the dynamic control regime. A stable carbonation conversion ratio of 52.0% was hold in the product layer control regime after 10 cycles. Also, the control sample Ca80Mn10Zr10‐R showed a quicker shift from the dynamic control to product control stage within 30 s in comparison with commercial CaCO_3_, indicating that the bimetallic co‐doping indeed boosted the carbonation rate. After 10 cycles, the carbonation conversion of Ca80Mn10Zr10‐R dropped from 42.5% to 40.1% in the product layer control regime, and yet no any changes in conversion rate were observed in the dynamic control regime. These results further confirmed that the 3DOM structure was able to exclude the dynamic limitation of CO_2_ diffusion from the external surface into the interior of CaO skeletons. The redshift of carbonation rate peaks shown in Figure 6c,d originated from the decrease in active sites of carbonation reaction on the surface of 3DOM CaO skeletons.

According to the Arrhenius formula of *k* = *A*exp(‐*E*/*RT*), the activation energies (*E*_a_) of calcination and carbonation reactions under different temperatures were calculated and shown in Figures S12–S19 (Supporting Information). Figure 6e,f showed, respectively, the activation energies of calcination and carbonation reactions functioned as the conversion ratio (α). It appeared that in the region of α = 0.1–0.6, all samples showed a uniform apparent activation energy of 181.5 ± 0.2 kJ mol^−1^, highly consistent with the results reported in literature.^[^ ^21^ ^]^ The results showed that both 3DOM structure and Mn, Zr co‐incorporation did not affect the decomposition kinetics. Interestingly, the apparent activation energy of carbonation reaction increased gradually with the α value located in the region of 0.05–0.35 due to the simultaneous occurrence of calcination and carbonation reactions at a high conversion ratio. The apparent activation energy of 3DOM Ca100‐350 was highly comparable with that of commercial CaCO_3_, indicating that the 3DOM structure did not alter the carbonation reaction kinetics_._ The apparent activation energy at the reaction fraction of 0.05 was equal to ≈31.0 kJ mol^−1^ for commercial CaCO_3_, ≈28.4 kJ mol^−1^ for 3DOM Ca100‐350, ≈24.4 kJ mol^−1^ for Ca80Mn10Zr10‐R and 26.3 kJ mol^−1^ for 3DOM Ca80Mn10Zr10 (Figure 6f). Notably, the apparent activation energy of 3DOM Ca80Mn10Zr10 and Ca80Mn10Zr10‐R was significantly lower than those of 3DOM Ca100‐350 and commercial CaCO_3_, suggesting that the introduction of inert components reduced the activation energy of carbonation reaction.

### 2.4 DFT Calculations

**Figure 7:**
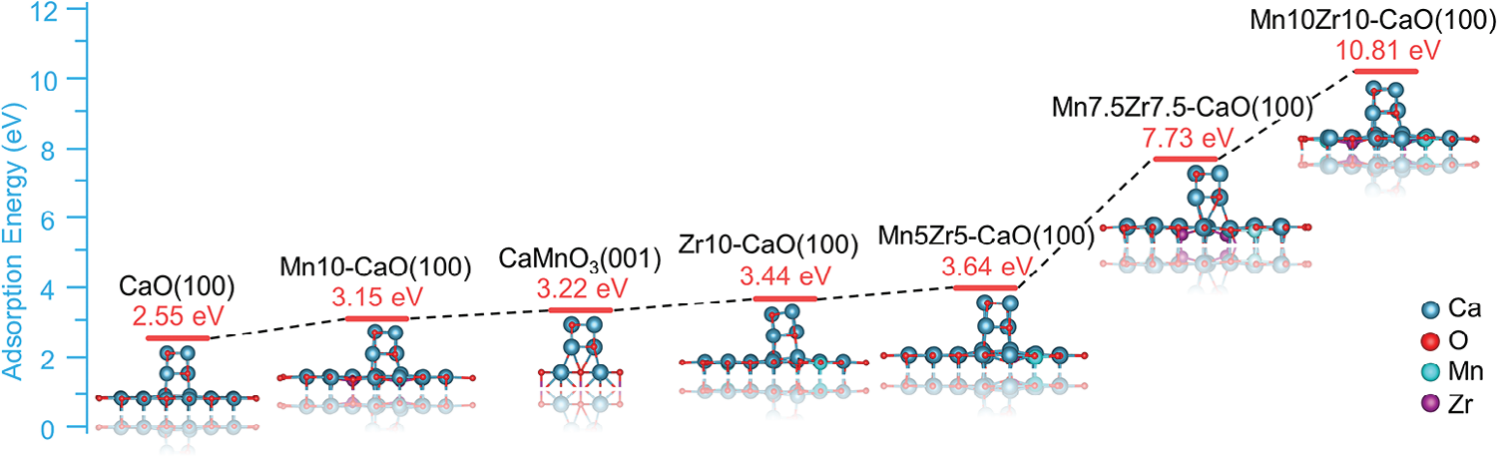
Adsorption energy of CaO nanoclusters on the metal‐doped CaO (100) and CaMnO_3_ (001) planes.

Understanding the sintering of Ca‐based materials in a molecular level, it was generally caused by the migration and agglomeration of Ca atoms under high temperature.^[^ ^22^ ^]^ The above‐mention characterization results revealed that a majority of inert Mn and Zr components were doped into the crystal lattice of 3DOM CaCO_3_ skeletons. With the high Mn doping content, a small amount of CaMnO_3_ NPs was formed to spatially separate CaO/CaCO_3_ NPs. Owing to the thermal instability of CaMnO_3_ that was self‐decomposed into CaO and MnO_2_ at 900 °C,^[^ ^23^ ^]^ the physical segmentation played a minor role in improving the cyclic stability of Ca‐based energy storage materials under the harsh conditions of 800 °C. A possible main contributor was the strong interfacial interaction between the Mn, Zr‐doped CaO materials and adjacent CaO NPs, making the CaO migration more difficult. Therefore, the density functional theory (DFT) calculation was used to figure out the interfacial interaction. A series of Mn/Zr‐doped CaO and CaMnO_3_ models shown in Figures S20–S27 (Supporting Information) were built to calculate the adsorption energies of [Ca_4_O_4_] clusters on the relatively stable (100) plane of CaO, Mn10‐CaO, Zr10‐CaO, Mn5Zr5‐CaO and Mn10Zr10‐CaO and on the (001) plane of CaMnO_3_. The DFT calculation results are shown in **Figure** **7**. The *E*_ad_ value was equal to 2.55 eV on the CaO (100) plane, 3.15 eV on the Mn10‐CaO (100) plane, 3.22 eV on the CaMnO_3_ (001) plane, 3.44 eV on the Zr10‐CaO (100) plane, 3.64 eV on the Mn5Zr5‐CaO (100) plane, 7.73 eV on the Mn7.5Zr7.5‐CaO (100) plane, and 10.81 eV on the Mn10Zr10‐CaO (100) plane. As expected, the adsorption energy of [Ca_4_O_4_] clusters on the CaMnO_3_ (001) plane was as low as 3.22 eV, comparable with those on the Mn10‐CaO (100) and Zr10‐CaO (100) planes, indicating that the interfacial interaction was very weak. It confirmed further that the enhanced cyclic stability was not contributed by the formation of CaMnO_3_. Amazingly, the Mn and Zr co‐incorporation enabled the giant improvement of adsorption energy for [Ca_4_O_4_] clusters. *E*_ad_ increased sharply with the Mn and Zr contents. Especially in the case of 20% molar doping amount, *E*_ad_ was substantially enhanced to 10.81 eV, showing the strong interfacial adsorption of the Mn10Zr10‐CaO (100) plane for [Ca_4_O_4_] clusters. The DFT calculation results were basically consistent with the above‐mention performance results shown in Figure 5, which showed that the cyclic performance of the materials follows the order of CaO < 3DOM Ca90Mn10 ≈ 3DOM Ca90Zr10 < 3DOM Ca90Mn5Zr5 < 3DOM Ca85Mn7.5Zr7.5 < 3DOM Ca80Mn10Zr10. It was thus concluded that the co‐incorporation of Mn and Zr into the crystal lattice of 3DOM CaCO_3_ skeletons generated a strong interfacial affinity to CaO, effectively suppressing the sintering and agglomeration of CaO nanoparticles at high temperatures. The strong chemical interaction made the thickness of 3DOM Ca‐based skeleton decrease greatly with increasing Zr and Mn.

## 3 Conclusion

In summary, a series of novel 3DOM Ca‐based materials with high energy storage density and excellent stability were successfully synthesized via a combined strategy of templating method with in situ co‐precipitation. The 3DOM structure featured with hierarchically mesoporous/macroporous skeletons further improved the solar absorptance of the Ca‐based materials. Moreover, it had a larger specific surface area, facilitating the CO_2_ diffusion and penetration to kinetically boost the calcination and carbonation reactions. The characterization studies and DFT calculations revealed that the co‐incorporation of Mn and Zr into the crystal lattice of CaO offered a strong interfacial affinity to prevent CaO NPs from sintering and agglomeration. As a result, the optimal Ca‐based material, 3DOM Ca80Mn10Zr10, exhibited a high average solar light absorption of 74.1%, a high initial energy storage density of 1706.4 kJ mol^−1^, and excellent cyclic stability performance. It gave a low energy storage density loss of 6.0% after 125 cycles. This work not only offered more options for designing Ca‐looping materials in the future but also demonstrated a promising way to utilize solar energy directly.

## Conflict of Interest

The authors declare no conflict of interest.

## Supporting information



Supporting Information

## Data Availability

The data that support the findings of this study are available from the corresponding author upon reasonable request.
