# Supplementary material for: 3D Ordered Macroporous Mn, Zr‐Doped CaCO3 Nanomaterials for Stable Thermochemical Energy Storage
Source: Adv Sci (Weinh). 2024 Dec 16;12(6):2412082. doi: 10.1002/advs.202412082 (PMC11809404; doi:10.1002/advs.202412082)
Supplement: Supplementary file 1 — Supporting Information [file ADVS-12-2412082-s001.docx]

**Supporting Information**

**Three-Dimensionally Ordered Macroporous Mn, Zr-doped CaCO_3_ Nanomaterials for Stable Thermochemical Energy Storage**

*Han Li^1^, Jinfeng Lin^1^, Jianze Wu^2^, Jiashun Wang^1^, Pengzhao Wang^3,^*, Guojian Kang^1^, Shuping Huang^2^, Mingkai Fu^4^, Jinjia Wei^5^, Zhengxin Ding^1,^*, and Jinlin Long^1,^**

^1^ State Key of Photocatalysis on Energy and Environment, Fuzhou University, Fuzhou, 350116, P. R. China.

Email: zxding@fzu.edu.cn (Z. D); jllong@fzu.edu.cn (J. L)

^2^ College of Chemistry, Fuzhou University, Fuzhou, 350116, P. R. China.

^3^ College of Chemical Engineering, Fuzhou University, Fuzhou, 350116, P. R. China.

Email: wpz@fzu.edu.cn

^4^ Institute of Electric Engineering, Chinese Academy of Sciences, Beijing, 100190, P. R. China.

^5^ College of Chemical Engineering, Xi’an Jiaotong University, Xi’an, 710049, P. R. China.

**1 Experimental section**

**1.1 Chemicals**

Sodium dodecyl sulfate (SDS, 99%, Shanghai Aladdin Biochemical Technology Co.), styrene (C_8_H_8_, AR, Shanghai McLean Biochemical Technology Co.), potassium persulfate (KPS, 99.99%, Shanghai Aladdin Biochemical Technology Co.), calcium acetate monohydrate (C_4_H_6_CaO_4_•H_2_O, AR, Sinopharm Chemical Reagent Co.), manganese acetate tetrahydrate (MnC_4_H_8_O_4_•4H_2_O, AR, Shanghai Aladdin Biochemical Technology Co.), zirconium acetate (C_8_H_12_O_8_Zr, 99.99%, Shanghai McLean Biochemical Technology Co.), ethanol absolute (EtOH, AR, Sinopharm Chemical Reagent Co.), sodium hydroxide (NaOH, AR, Sinopharm Chemical Reagent Co.), sodium hydrogen carbonate (NaHCO_3_, AR, Sinopharm Chemical Reagent Co.), and ultrapure water.

**1.2 Synthesis of polystyrene sphere (PS) templates**

Monodisperse colloidal PSs with a diameter of 160 nm, 350 nm, and 450 nm were synthesized using a modified method reported in the literature.^[1]^ The preparation of 350 nm PS templates was as follows. Typically, 40 mL of styrene (treated with 40 mL of 10 wt.% NaOH) were added to a 500 mL three-neck flask filled with 250 mL of water and 150 mL of anhydrous ethanol. Then, the above mixture was stirred magnetically at 70 ℃ for 30 minutes. Subsequently, 0.6 g of K_2_S_2_O_8_, 0.18 g of NaHCO_3_, and 0.45 g of sodium dodecyl sulfate (DBS) were added to the flask. Monodisperse colloidal PSs were obtained by stirring for 20 h. Three-dimensionally ordered PS templates were obtained by centrifugation at 3k rpm for 9 h.

The preparation of 160 nm PS templates was as follows. Typically, 30 mL of styrene (treated with 30 mL of 10 wt.% NaOH) was added to a 500 mL three-neck flask filled with 70 mL of water and 30 mL of anhydrous ethanol. Then, the above mixture was stirred magnetically at 70 ℃ for 30 minutes. Subsequently, 0.25 g of K_2_S_2_O_8_, 0.08 g of NaHCO_3_, and 0.4 g of sodium dodecyl sulfate (DBS) were added to the flask. Monodisperse colloidal PSs were obtained by stirring for 20 h. Three-dimensionally ordered PS templates were obtained by centrifugation at 3k rpm for 9 h.

The preparation of 450 nm PS templates was as following. Typically, 90 mL of styrene (treated with 90 mL of 10 wt.% NaOH) was added to a 500 mL three-neck flask filled with 380 mL of water. Then, the above mixture was stirred magnetically at 70 ℃ for 30 minutes. Subsequently, 0.4 g of K_2_S_2_O_8_ and 0.15 g of NaHCO_3_, were added to the flask. Monodisperse colloidal (PS) was obtained by stirring for 20 h. Three-dimensionally ordered PS templates were obtained by centrifugation at 3 krpm for 9 h.

**1.3 Synthesis of 3D ordered macroporous Ca-based materials**

A series of 3D ordered macroporous Ca-based materials with Mn and Zr dopants were synthesized by a modified procedure reported in literature^[2]^. Calcium acetate monohydrate, manganese acetate tetrahydrate, and zirconium acetate were firstly dissolved in 10 mL of ethanol aqueous solution (7 mL water together with 3 mL ethanol) to obtain the precursor solution. After the as-prepared PS templates were heated and pretreated at 100 ^o^C for 15 min, a piece of colloidal PS templates were taken and immersed into the above precursor solution for 30 min. The resultant solid mixture was subjected to the vacuum filtration and to be dried at 60 ^o^C for 24 h, subsequently it was immersed into 30 wt.% oxalic acid solution for 30 min, along with the vacuum filtration. Finally, the resultant solid powder was calcined at 300 ^o^C for 2 h and 500 ^o^C for 2 h under the air atmosphere with a 1 k min^-1^ heating rate.

**1.4 Material characterizations**

The Mn and Zr contents of the samples were determined by ICP analysis (PerkinElmer Avio 200 ICP-OES). The X-ray diffraction (XRD) characterization was performed on a Bruker D8 Advance diffractometer (CuK radiation, λ = 1.5406 μm) operated at 40 kV and 40 mA with a data acquisition range of 5 - 80^o^ (2θ). The shape and morphology were characterized by Hitachi SU8010 high-resolution field emission scanning electron microscopy (SEM) and by Thermofiher Scientific (FEI) Talos F200S transmission electron microscope (TEM). The atomic resolution (mapping) images were taken in high-angle annular dark-field (HAADF) mode. The spherical aberration-corrected transmission characterization was performed with two Themis Z-type spherical aberration-corrected transmission electron microscopes with operating voltages of 300 kV. The X-ray photoelectron (XPS) spectra were acquired on a Thermo Scientific Escalab 250Xi X-ray photoelectron spectrometer equipped with a monochromatic Al-Kα radiation source (h = 1486.6 eV), where all the spectra were corrected to the C1s peak at 284.6 eV for surface deposited carbon. The BET surface area and pore size were measured with an N_2_ adsorption/desorption isotherm at 77 K on a Micromeritiecs 3Flex instrument.

**1.5 Solar absorptance testing**

The samples were pressed into circular tablets, and then the reflectance was measured in the wavelength range of 200-2500 nm on a UV-Vis-NIR spectrometer (Carry 5000, Agilent Technologies) with an integrating sphere, and BaSO_4_ was used as the reference sample of the calibration instrument. The solar absorptance was done by calculating the weighted product of the absorbance and the standard solar spectrum (AM 1.5 Direct). The following equation gave the mathematical expression for the average solar absorptance:

$A=\frac{\int_{200 nm}^{2500 nm} a(\lambda)I(\lambda)d\lambda}{\int_{200 nm}^{2500 nm} I(\lambda)d\lambda}$ (1)

Where A is the average solar absorptance, α (λ) = (1-R (λ)) and I (λ) are spectral absorptivity and solar spectral irradiance under the condition of AM 1.5D, respectively. λ is the wavelength.

**1.6 Cyclic performance testing**

The cycling performance was studied using a thermogravimetric analyzer (TGA, Jinyi). A small amount (< 10 mg) of the sample was heated to 800 ℃ at a nitrogen flow rate of 100 mL min^-1^ and a heating rate of 20 ℃ min^-1^. Once the reaction temperature was stabilized, the 80 mL min^-1^ N_2_ stream in the atmosphere was changed to the 80 mL min^-1^ CO_2_ stream to carbonize the sample. The carbonization was carried out for 7 min. Subsequently, the CO_2_ flow was stopped and the 80 mL min^-1^ CO_2_ flow was changed to the 80 mL min^-1^ N_2_ flow. The calcination was carried out for 4 min. Repeating the calcination and carbonation steps with the desired number of cycles was to determine the thermochemical stablity of the sample. The change in adsorbent weight was continuously monitored and recorded throughout the operation process.

The cyclic properties of materials were evaluated using effective conversion and energy storage density. The formula was as following:

$X_{e,N}=\frac{m_{carN}-m_{calN-1}}{m_{0}}\cdot\frac{M_{CaO}}{M_{{CO}_{2}}}$ (2)

Where X_e,N_ are the effective conversion rate of the N^TH^ time; m_carN_ and m_calN-1_ are the mass after the N^TH^ carbonation and the mass after the N-1 calcination, respectively; m_0_ is the mass of the initial CaO; M_CaO_ and MCO_2_ are the relative molar mass of CaO and CO_2_, respectively.

$E_{e,N}=X_{e,N}\cdot\frac{\Delta H_{r}}{M_{CaO}}$ (3)

Where E_e, N_ is the energy storage density at the N^TH^ time, X_e, N_ is the effective conversion rate at the N^TH^ time, △H_r_ is the molar enthalpy of the calcium carbonate decomposition reaction (178 kJ mol^-1^), and M_CaO_ is the relative molar mass of CaO.

**1.7 Reaction kinetics**

After the mass action law proposed by Guldberg and Waage, Van't Hoff proposed the concept of reaction order on this basis. Therefore, the classical isothermal kinetic formula of homogeneous reaction is basically completed, which can be expressed as:

$\frac{dc}{dt}=k(T)f(c)$ (4)

Where *c* is the product concentration, *k* is the rate constant, and $f(c)$ is the correlation reaction function. However, the concentration *c* in the formula is not suitable in the heterogeneous reaction, so an optimization is made to replace the concentration (c) with the reaction fraction (α) to satisfy the kinetics of the heterogeneous reaction.

$\frac{dc}{dt}=k\left( T \right)f\left( \alpha\right)$ (5)

S. Arrhenius of Sweden proposed the classic Arrhenius formula which is the relationship between reaction rate and temperature in 1889.

$k=Aexp\left( -\frac{E}{RT} \right)$ (6)

Generally, $f\left( \alpha\right)$ = (1-α)^n^ is used to represent the reaction function, and the basic equation of isothermal heterogeneous reaction kinetics can be derived:

$ln \frac{d\alpha}{dt}=ln A-\frac{E}{RT}+nln \left( 1-\alpha\right)$ (7)

Where A and E are the empirical constants of the specific reaction, R is the molar gas constant, and T is the specific temperature of the reaction.

The reaction kinetics of 3DOM Ca100-350, commercial CaCO_3_, 3DOM Ca80Mn10Zr10, and Ca80Mn10Zr10-R were studied with thermogravimetric analyzer (TGA, ZXF-B Jinyi). A small amount of (5±1 mg) samples were placed in an aluminum tray. The calcination conditions are as follows. Under the 100 mL min^-1^ N_2_ flow, the temperature was heated to 700, 725, 750, 775, and 800 ^o^C for 10 min at the rate of 40 ^o^C min^-1^. The reaction temperature is stabilized, The N_2_ flow of 100 mL min^-1^ was reduced to 20 mL min^-1^, and 80 mL min^-1^ CO_2_ flow was added to the N_2_ flow to carbonate the sample for 15 min. Subsequently, the CO_2_ flow was stopped, N_2_ flow was increased to 100 mL min^-1^, and calcined for 30 min. Changes in the weight of the adsorbent are continuously monitored and recorded throughout the operation. The carbonation conditions are the same as above, the atmosphere is changed to CO_2_.

**1.8 Density functional theory calculations**

A cubic CaO cluster consisting of four Ca atoms and four O atoms was built for the structural optimization calculations. Then, the CaO (100) surface were chosen as they were considered to be the most stable surface.^[3]^ Four layers of the CaO (100) surface with a vacuum layer of 15 Å thickness were cleaved from a single CaO cell. The two layers at the bottom were fixed and the other layers were relaxed to finally optimize the structure through the DFT calculation. The Mn10Zr10-CaO (100) surface was constructed in the same manner as described above, where 7 Ca atoms were replaced with Mn atoms and 7 Ca atoms were replaced with Zr atoms. The Mn7.5Zr7.5-CaO (100) surface was constructed in the same way as above, where 5 Ca atoms were replaced by Mn atoms and 5 Ca atoms were replaced by Zr atoms. The Mn5Zr5-CaO (100) surface was constructed in the same way as above, where 4 Ca atoms were replaced by Mn atoms and 4 Ca atoms were replaced by Zr atoms. The Mn10-CaO (100) surface was constructed in the same way as above, where 7 calcium atoms were replaced by Mn atom. The Zr10-CaO (100) surface was constructed in the same way as above, where 7 calcium atoms were replaced by Zr atom. The optimized CaO clusters described above were added to the above (100) surface and CaMnO_3_ (001) surface to simulate the adsorption of CaO on the material. The structural optimization calculations were performed. The adsorption energy was used for evaluation. The adsorption energy was calculated as follows:^[4]^

$E_{ad}=E_{adsorbate + surface}-E_{adsorbate}-E_{surface}$ (8)

Where, E_adsorbate + surface_  is the total system energy of adsorbate on the material surface, E_adsorbate_ is adsorbed energy and E_surface_ is surface energy.

**2. Figure S1 to 29**

**Figure S1**. SEM image of (**a**) 160 nm PS templates and (**b**) 450 nm PS templates. Three-dimensional ordered PS templates can be obtained by centrifugal self-assembly.

**Figure S2**. SEM image of 350 nm PS templates at different magnifications. Three-dimensional ordered PS templates can be obtained by centrifugal self-assembly.

**Figure S3**. SEM image of (**a**) 3DOM Ca100-160 and (**b**) -450 samples.

**Figure S4**. SEM image of commercial CaCO_3_ at different magnifications. The sample was obtained from Sinopharm Chemical Reagent Co.


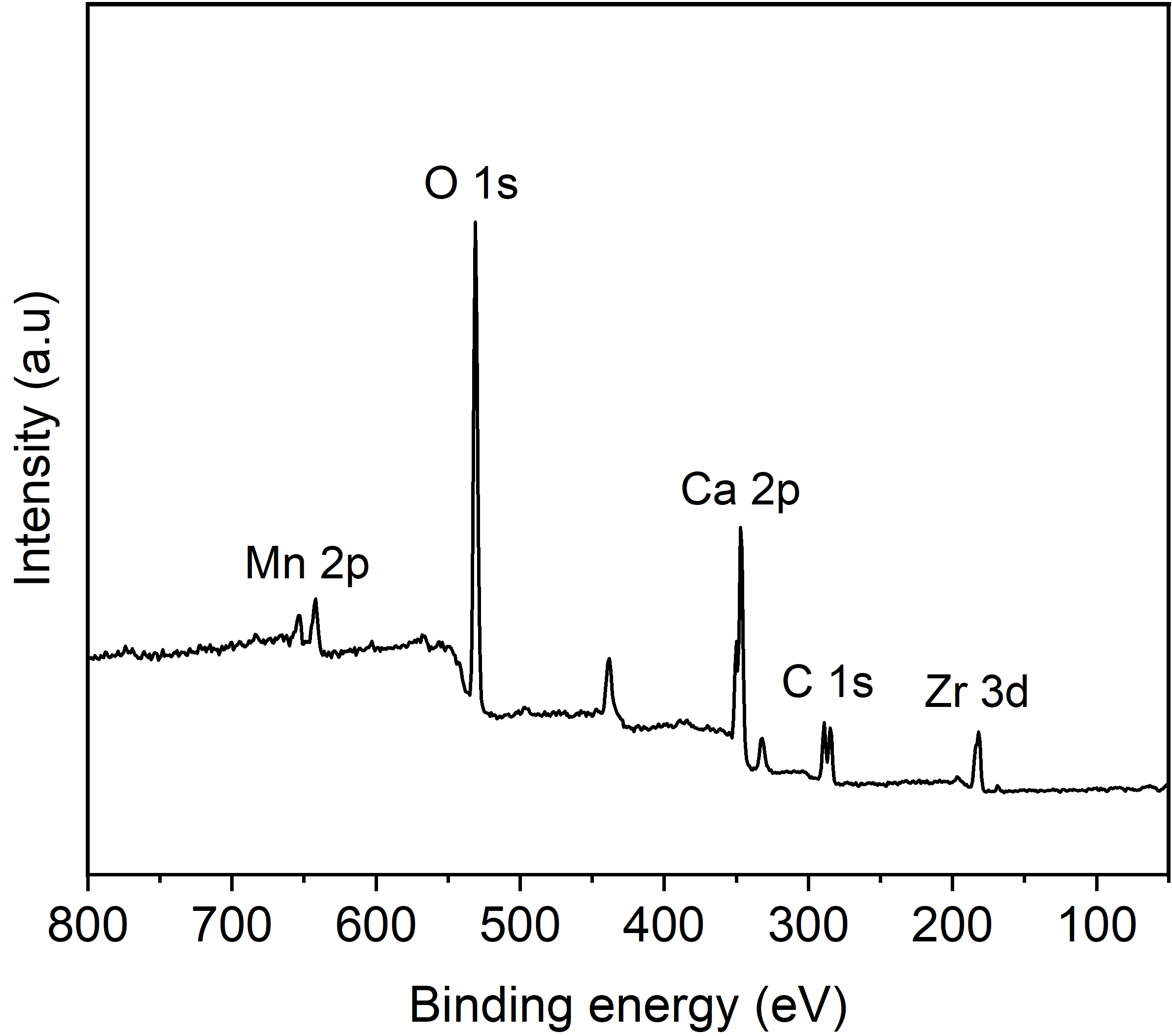


**Figure S5** (**a**) UV-Vis-NIR DRS spectra of 3DOM Ca100-350 and commercial CaCO_3_, 3DOM Ca80Mn10Zr10, and Ca80Mn10Zr10-R. (**b**) UV-Vis-NIR DRS spectra of 3DOM Ca90Mn5Zr5, and Ca90Mn5Zr5-R. (**c**) UV-Vis-NIR DRS spectra of 3DOM Ca90Mn7.5Zr7.5, and Ca90Mn7.5Zr7.5-R. (**d**) UV-Vis-NIR DRS spectra of Ca90Mn5Zr5-R, Ca90Mn7.5Zr7.5-R, and Ca80Mn10Zr10-R.

**Figure S6**. TGA traces of (**a**) 3DOM Ca100-350 and (**b**) commercial CaCO_3_.

**Figure S7**. TGA traces of (**a**) 3DOM Ca90Mn10 and (**b**) Ca90Mn10-R.

**Figure S8**. TGA traces of (**a**) 3DOM Ca90Zr10 and (**b**) Ca90Zr10-R.

**Figure S9**. TGA traces of (**a**) 3DOM Ca90Mn5Zr5 and (**b**) Ca90Mn5Zr5-R.

**Figure S10**. TGA traces of (**a**) 3DOM Ca85Mn7.5Zr7.5 and (**b**) Ca85Mn7.5Zr7.5-R.

**Figure S11**. TGA trace of 3DOM Ca80Mn10Zr10.

In Figure S6-11, "Weight" in y axis refers to the change in mass of the sample and "Time" in x axis refers to the time scale. Test conditions for the TGA trace: The sample was heated to 800 ℃ at a nitrogen flow rate of 100 mL min^-1^ and a heating rate of 20 ℃ min^-1^. Once the reaction temperature was stabilizesd, the 80 mL min^-1^ N_2_ stream in the atmosphere was changed to the 80 mL min^-1^ CO_2_ stream to carbonize the sample. The carbonization step was carried out for 7 min. Subsequently, the CO_2_ flow was stopped and the 80 mL min^-1^ CO_2_ flow of was changed to the 80 mL min^-1^ N_2_ flow. The calcination step was carried out for 4 min. Repeating the calcination and carbonation steps were repeated with 125 cycles, and the change in adsorbent weight was continuously monitored and recorded throughout the operation process.

**Figure S12**. (**a**) TG curves, (**b**) DTG curves, (**c**) ln(dα/dt)-T^-1^ diagram at different α values, and (**d**) E_a_ diagram at different α values of commercial CaCO_3_ at different temperatures with calcination reactions.

**Figure S13**. (**a**) TG curves, (**b**) DTG curves, (**c**) ln(dα/dt)-T^-1^ diagram at different α values, and (**d**) E_a_ diagram at different α values of 3DOM Ca100-350 at different temperatures with calcination reactions.

**Figure S14**. (**a**) TG curves, (**b**) DTG curves, (**c**) ln(dα/dt)-T^-1^ diagram at different α values, and (**d**) E_a_ diagram at different α values of CaMn10Zr10-R at different temperatures with calcination reactions.

**Figure S15.** (**a**) TG curves, (**b**) DTG curves, (**c**) ln(dα/dt)-T^-1^ diagram at different α values, and (**d**) E_a_ diagram at different α values of 3DOM CaMn10Zr10 at different temperatures with calcination reactions.

**Figure S16**. (**a**) TG curves, (**b**) DTG curves, (**c**) ln(dα/dt)-T^-1^ diagram at different α values, and (**d**) E_a_ diagram at different α values of commercial CaCO_3_ at different temperatures with carbonation reactions.

**Figure S17**. (**a**) TG curves, (**b**) DTG curves, (**c**) ln(dα/dt)-T^-1^ diagram at different α values, and (**d**) E_a_ diagram at different α values of 3DOM C100-350 at different temperatures with carbonation reactions.

**Figure S18**. (**a**) TG curves, (**b**) DTG curves, (**c**) ln(dα/dt)-T^-1^ diagram at different α values, and (**d**) E_a_ diagram at different α values of Ca80Mn10Zr10-R at different temperatures with carbonation reactions.

**Figure S19**. (**a**) TG curves, (**b**) DTG curves, (**c**) ln(dα/dt)-T^-1^ diagram at different α values, and (**d**) E_a_ diagram at different α values of 3DOM Ca80Mn10Zr10 at different temperatures with carbonation reactions.

**Figure S20**. Optimized structure models of CaO cluster.

**Figure S21**. (**a**) Optimized structures of CaO (100) surface and (**b**) Optimized structures of CaO cluster adsorbed on periodic CaO (100) surface.

**Figure S22**. (**a**) Optimized structures of Mn10-CaO (100) surface and (**b**) Optimized structures of CaO cluster adsorbed on periodic Mn10-CaO (100) surface.

**Figure S23**. (**a**) Optimized structures of CaMnO_3_ (001) surface and (**b**) Optimized structures of CaO cluster adsorbed on periodic CaMnO_3_ (001) surface.

**Figure S24**. (**a**) Optimized structures of Zr10-CaO (100) surface and (**b**) Optimized structures of CaO cluster adsorbed on periodic Zr10-CaO (100) surface.

**Figure S25**. (**a**) Optimized structures of Mn5Zr5-CaO (100) surface and (**b**) Optimized structures of CaO cluster adsorbed on periodic Mn5Zr5-CaO (100) surface.

**Figure S26**. (**a**) Optimized structures of Mn7.5Zr7.5-CaO (100) surface and (**b**) Optimized structures of CaO cluster adsorbed on periodic Mn7.5Zr7.5-CaO (100) surface.

**Figure S27**. (**a**) Optimized structures of Mn10Zr10-CaO (100) surface and (**b**) Optimized structures of CaO cluster adsorbed on periodic Mn10Zr10-CaO (100) surface.


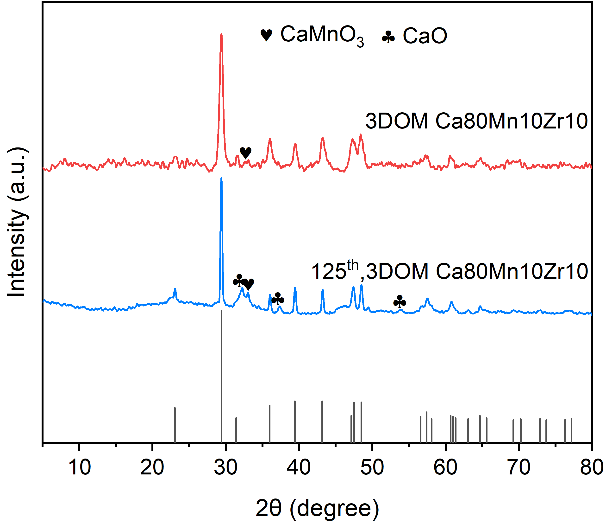


**Figure S28**. XRD of fresh and 125^th^ cycled 3DOM Ca80Mn10Zr10.


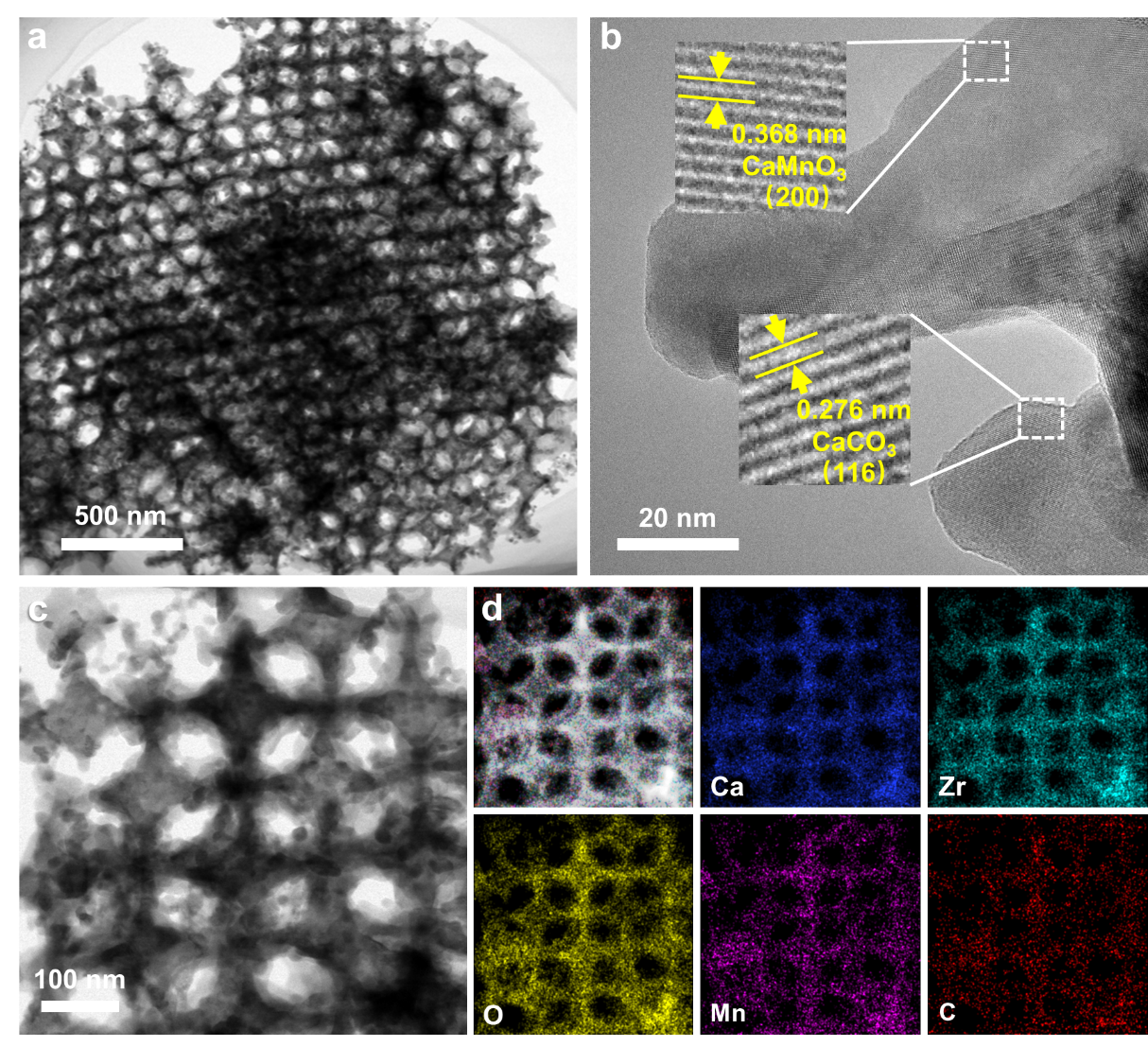


**Figure S29**. (**a**, **b**) TEM and HRTEM images of 125^th^ cycle 3DOM Ca80Mn10Zr10. (**c**, **d**) HAADF-STEM and EDX elemental mapping images of 125^th^ cycled 3DOM Ca80Mn10Zr10.

Compared to the SEM image of fresh 3DOM Ca80Mn10Zr10 shown in **Figure** **2k**, it is clearly observed that the mesopores of the used 3DOM Ca80Mn10Zr10 are partly blocked by CaCO_3_ nanoparticles, which are dropped away from the 3DOM skeleton during the repeated calcination and carbonation reactions at 800 ℃. It can be thus concluded that the slow decay of thermochemical energy storage is mainly ascribed to the channel block. It tells us to further enhance the stability by improving the mass transfer rates of the calcination and carbonation reactions in the future.

**3. Table S1 to 6**

**Table S1**. Mn and Zr content of different samples.

| Samples | Mn content  [wt%] | Zr content  [wt%] | Ca: Mn: Zr  (molar ratio) |
| --- | --- | --- | --- |
| 3DOM Ca90Mn5Zr5 | 8.5 | 10.6 | 88.1：6.8：5.1 |
| 3DOM Ca85Mn7.5Zr7.5 | 8.6 | 17.0 | 84.4：7.1：8.5 |
| 3DOM Ca80Mn10Zr10 | 13.5 | 20.6 | 78.1：11.4：10.5 |

**Table S2**. Specific surface areas and pore volume of different samples.

| Samples | BET surface area  [m^2^ g^-1^] | Pore volume  [m^3^ g^-1^] |
| --- | --- | --- |
| 3DOM Ca100-160 | 9.70 | 0.103 |
| 3DOM Ca100-350 | 16.49 | 0.158 |
| 3DOM Ca100-450 | 11.78 | 0.074 |
| 3DOM Ca90Mn5Zr5 | 49.82 | 0.384 |
| 3DOM Ca85Mn7.5Zr7.5 | 56.87 | 0.455 |
| 3DOM Ca80Mn10Zr10 | 65.45 | 0.471 |

The pore volume is the combined micropores/mesopores larger than 1.7 nm.

**Table S3**. The crystal cell parameters of as-synthesized 3DOM CaCO_3_ samples.

| Crystal cell parameters | Ca100-350 | Ca90Mn5Zr5 | Ca85Mn7.5Zr5 | Ca80Mn10Zr10 |
| --- | --- | --- | --- | --- |
| *d*_(104)_ [Å] | 3.0416 | 3.0376 | 3.0315 | 3.0324 |
| *d*_(113)_ [Å] | 2.2870 | 2.2848 | 2.2814 | 2.2826 |
| *A* [Å] | 4.992 | 4.989 | 4.982 | 4.985 |
| *C* [Å] | 17.118 | 17.087 | 17.040 | 17.039 |
| V [Å^3^] | 369.43 | 368.32 | 366.28 | 366.70 |

**Table S4**. Thermochemical cycling properties of different samples.

| Samples | Initial convers-ion | Initial energy storage density  [kJ kg^-1^] | Cycle number | Convers-ion after cycle | Energy storage density after cycle  [kJ kg^-1^] | Attenua-tion |
| --- | --- | --- | --- | --- | --- | --- |
| Commercial CaCO_3_ | 70.4% | 2240.2 | 50 | 10.9% | 346.6 | 84.5% |
| 3DOM  Ca100-350 | 65.9% | 2096.5 | 50 | 38.4% | 1221.1 | 41.7% |
| 3DOM Ca90Zr10 | 77.7% | 2470.2 | 60 | 54.3% | 1726.7 | 30.1% |
| Ca90Zr10-R | 58.4% | 1857.1 | 60 | 42.1% | 1338.8 | 27.4% |
| 3DOM Ca90Mn10 | 67.1% | 2136.9 | 60 | 50.8% | 1615.4 | 24.4% |
| Ca90Mn10-R | 60.3% | 1917.5 | 60 | 39.8% | 1265.6 | 33.9% |
| 3DOM Ca90Mn5Zr5 | 74.6% | 2371.3 | 60 | 64.6% | 2054.3 | 13.4% |
| Ca90Mn5Zr5-R | 65.6% | 2087.4 | 60 | 49.5% | 1574.1 | 24.6% |
| 3DOM Ca85Mn7.5Zr7.5 | 67.9% | 2159.2 | 60 | 61.7% | 1960.1 | 11.9% |
| Ca85Mn7.5Zr7.5-R | 61.1% | 1943.0 | 60 | 49.9% | 1586.8 | 18.3% |
| 3DOM Ca80Mn10Zr10 | 53.7% | 1706.4 | 60 | 52.4 | 1666.9 | 2.3% |
|  |  |  | 125 | 51.5 | 1603.9 | 6.0% |
| Ca80Mn10Zr10-R | 42.5% | 1350.2 | 60 | 36.4% | 1158.5 | 14.2% |

**Table S5**. Representative works on Ca-based thermochemical energy storage.

| Samples | Initial energy storage density  [kJ kg^-1^] | Cycle number | Energy storage density after cycle  [kJ kg^-1^] | Attenuation | Conditions  (carbonation/  calcination) |
| --- | --- | --- | --- | --- | --- |
| This work | 1706 | 125 | 1604 | 6.0% | 800 ℃, 7 min/800 ℃, 4 min |
| Mn, Mg/CaO^[5]^ | 1520 | 100 | 1460 | 4.0% | 800 ℃, 10 min/800 ℃, 8 min |
| Ti, Mg, Al/CaO^[6]^ | 1175 | 100 | 1069 | 9.0% | 750 ℃, 10 min/800 ℃, 5 min |
| Mn, Al/CaO^[7]^ | 1143 | 20 | 1017 | 11.1% | 800 ℃, 15 min/800℃, 15 min |
| Mg, Zn/CaO^[8]^ | 2662 | 25 | 1900 | 28.6% | 850 ℃, 15 min/880℃, 20 min |
| Mn, Mg/CaO^[9]^ | 1960 | 25 | 1648 | 15.9% | 800 ℃, 10 min/800℃, 14 min |
| SiC, Mn/CaO^[4]^ | 2384 | 30 | 1367 | 42.7% | 800 ℃, 10 min/950℃, 10 min |
| Mn, Fe/CaO^[10]^ | 1450 | 60 | 1400 | 3.3% | 700 ℃, 10 min/700℃, 15 min |
| Mg/CaO^[11]^ | 2034 | 30 | 1907 | 6.3% | 650 ℃, 20 min/900 ℃, 5 min |
| Fe, Mn/CaO^[12]^ | 1684 | 20 | 1589 | 5.7% | 600 ℃, 20 min/850 ℃, 10 min |
| Samples | Initial energy storage density  [kJ kg^-1^] | Cycle number | Energy storage density after cycle  [kJ kg^-1^] | Attenuation | Conditions  (carbonation/  calcination) |
| Mn, Fe/CaO^[13]^ | 2288 | 20 | 2129 | 7.0% | 700 ℃,15 min/700 ℃, 10 min |
| Mn, Fe/CaO^[14]^ | 636 | 100 | 556 | 12.5% | 750 ℃, 60 min/750 ℃, 60 min |
| Ce, La, Nd, Yb, Zr/CaO^[15]^ | 1748 | 20 | 1653 | 5.4% | 650 ℃, 30 min/850 ℃, 5 min |
| Na/CaO^[16]^ | 2161 | 50 | 1112 | 48.5% | 650 ℃, 20 min/800 ℃, 10 min |
| Zr/CaO^[17]^ | 2168 | 21 | 1951 | 10.0% | 675 ℃, 10 min/850 ℃, 10 min |
| SiO_2/_CaO^[18]^ | 2542 | 50 | 1176 | 53.7% | 850 ℃, 35 min/650 ℃, 40 min |
| SiC/CaO^[19]^ | 1120 | 40 | 482 | 57.0% | 884 ℃, 10 min/884 ℃, 20 min |
| Cu, Al/CaO^[20]^ | 694 | 20 | 601 | 13.4% | 750 ℃, 20 min/750 ℃, 10 min |
| Al/CaO^[21]^ | 2002 | 30 | 1748 | 12.7% | 650 ℃, 20 min/900 ℃, 10 min |
| Samples | Initial energy storage density  [kJ kg^-1^] | Cycle number | Energy storage density after cycle  [kJ kg^-1^] | Attenuation | Conditions  (carbonation/  calcination) |
| Ti, Ni/CaO^[22]^ | 1296 | 20 | 1267 | 2.2% | 750 ℃, 10 min/850 ℃, 5 min |
| Fe/CaO^[23]^ | 1629 | 40 | 622 | 61.8% | 860 ℃, 20 min/860 ℃, 10 min |

**Table S6**. Energy of different optimization models.

| Name | E_adsorbate + surface_  [eV] | E_surface_  [eV] | E_adsorbate_  [eV] |
| --- | --- | --- | --- |
| CaO (100) | -951.38 | -905.70 | -43.13 |
| Mn10-CaO (100) | -959.14 | -912.86 | -43.13 |
| CaMnO_3_ (001) | -327.70 | -281.36 | -43.13 |
| Zr10-CaO (100) | -987.32 | -940.75 | -43.13 |
| Mn5Zr5-CaO (100) | -980.74 | -933.97 | -43.13 |
| Mn7.5Zr7.5-CaO (100) | -992.35 | -941.49 | -43.13 |
| Mn10Zr10-CaO (100) | -1004.47 | -950.53 | -43.13 |

**Table S7.** Volumetric energy storage densities of Ca-based materials

| Sample | Density (g cm^-3^) | Initial volumetric energy storage density (kJ m^-3^) |
| --- | --- | --- |
| Commercial CaCO_3_ | 2.7445 | 6.1477ⅹ10^6^ |
| 3DOM Ca100-350 | 0.7454 | 1.5627ⅹ10^6^ |
| 3DOM Ca80Mn10Zr10 | 0.7484 | 1.2771ⅹ10^6^ |
| 3DOM Ca85Mn7.5Zr7.5 | 0.8185 | 1.7673ⅹ10^6^ |
| 3DOM Ca90Mn5Zr5 | 0.7847 | 1.8608ⅹ10^6^ |

**References:**

[1] Z. Chen, S. Wu, J. Ma, S. Mine, T. Toyao, M. Matsuoka, L. Wang, J. Zhang, *Angewandte Chemie International Edition* **2021**, 60, 11901.

[2] H. W. Yan, C. F. Blanford, B. T. Holland, W. H. Smyrl, A. Stein, *Chemistry of Materials* **2000**, 12, 1134.

[3] B. Galloway, B. Padak, *Fuel* **2017**, 197, 541.

[4] B. Li, Y. Li, Y. Dou, Y. Wang, J. Zhao, T. Wang, *Chemical Engineering Journal* **2021**, 423, 130305.

[5] H. Liu, Y. Li, J. Wei, *Chemical Engineering Journal* **2024**, 480, 147892.

[6] X. K. Tian, S. C. Lin, J. Yan, C. Y. Zhao, *Chemical Engineering Journal* **2022**, 450, 138142.

[7] X. Wang, X. Liu, H. Zheng, C. Song, K. Gao, C. Tian, N. Sun, Z. Jiang, *Solar Energy* **2023**, 251, 197.

[8] Y. Zhang, Y. Li, Y. Xu, F. Wang, Z. Wei, Y. Fang, C. Li, Z. He, *Journal of Energy Storage* **2023**, 66, 107447.

[9] H. Liu, J. Zhang, J. Wei, *Solar Energy Materials and Solar Cells* **2023**, 252, 112202.

[10] L. Teng, Y. Xuan, Y. Da, X. Liu, Y. Ding, *Energy Storage Materials* **2020**, 25, 836.

[11] M. A. Naeem, A. Armutlulu, Q. Imtiaz, F. Donat, R. Schäublin, A. Kierzkowska, C. R. Müller, *Nature Communications* **2018**, 9, 2408.

[12] C. Zhang, Y. Li, Z. He, J. Zhao, D. Wang, *Applied Catalysis B: Environmental* **2022**, 314, 121474.

[13] L. Yang, G. Huang, Z. Huang, *Journal of Energy Storage* **2021**, 43, 103236.

[14] L. Desage, T. D. Humphries, M. Paskevicius, C. E. Buckley, *Journal of Materials Chemistry A* **2024**, 12, 14721

[15] Y. Long, Q. Gu, C. Wang, X. Zhang, H. Liu, L. Liu, Z. Zhou, *Small* **2024**, 2406165.

[16] M. Krödel, L. Abduly, M. Nadjafi, A. Kierzkowska, A. Yakimov, A. H. Bork, F. Donat, C. Copéret, P. M. Abdala, C. R. Müller, *Advanced Functional Materials* **2023**, 33, 2302916.

[17] N. Mahinpey, D. Karami, *Catalysis Today* **2022**, 404, 237.

[18] C.-C. Li, U.-T. Wu, H.-P. Lin, *J. Mater. Chem. A* **2014**, 2, 8252.

[19] T. Richardson, R. K. Vijayaraghavan, P. J. McNally, M. V. Sofianos, *Journal of Alloys and Compounds* **2023**, 934, 167844.

[20] S. Xing, R. Han, Y. Wang, C. Pang, Y. Hao, X. Wu, C. Song, Q. Liu, *Microporous and Mesoporous Materials* **2022**, 337, 111923.

[21] A. Armutlulu, M. A. Naeem, H. J. Liu, S. M. Kim, A. Kierzkowska, A. Fedorov, C. R. Müller, *Advanced Materials* **2017**, 29, 1702896.

[22] S. J. Guo, X. K. Tian, J. Yan, S. H. Ju, C. Y. Zhao, *Journal of Materials Chemistry A* **2024**, 12, 14129.

[23] R. Anwar, R. K. Vijayaraghavan, P. J. McNally, M. M. Dardavila, E. Voutsas, M. V. Sofianos, *RSC Advances* **2023**, 13, 32523.
